# Characterization and Functional Evaluation of Carotenoids From *Haloarcula rubripromontorii* BS2

**DOI:** 10.1002/mbo3.70228

**Published:** 2026-02-05

**Authors:** Devika N. Nagar, Deepthi Das, Raviprasad Aduri, Judith Maria Braganca

**Affiliations:** ^1^ Department of Biological Sciences Birla Institute of Technology and Science, K K Birla Goa Campus Zuarinagar Goa India; ^2^ Department of Chemistry Birla Institute of Technology and Science, K K Birla Goa Campus Zuarinagar Goa India

**Keywords:** bacterioruberin, carotenoids, halophilic archaea, pigments

## Abstract

*Haloarcula rubripromontorii* BS2, an extremely halophilic archaeon was obtained from the solar salt pans of Goa, India. It grew luxuriantly on EHM medium with 25% NaCl with a bright orange pigmentation. This study aimed to extract and characterize the carotenoids from *Haloarcula rubripromontorii* BS2 and evaluate their antioxidant properties, biocompatibility and provide insight into their potential therapeutic applications. Preparative high‐performance liquid chromatography (HPLC) was employed to fractionate and separate the haloarchaeal carotenoids. Further detailed characterization using liquid chromatography‐mass spectrometry (LC‐MS), Raman spectroscopy and nuclear magnetic resonance (NMR) spectroscopy for each fraction confirmed the presence of C50 carotenoids primarily bacterioruberin, monoanhydrobacterioruberin, and their different isomeric forms. Our results indicate that these carotenoids are more stable in oil compared to solvents. 2,2‐Diphenyl‐1‐picrylhydrazyl (DPPH) and 2,2'‐azino‐bis(3 ethylbenzothiazoline‐6‐sulfonic acid (ABTS) assays resulted in an IC_50_ of 4.31 ± 0.07 µgmL^−1^ and 2.04 ± 0.02 µgmL^−1^ respectively, indicating their potential as excellent antioxidants. Haloarchaeal carotenoids were found to be biocompatible with human keratinocyte skin cells (HaCaT). C50 carotenoids from *Har. rubripromontorii* BS2 represent promising, eco‐friendly alternatives to synthetic antioxidants for use in high‐value cosmetic and dermatological applications.

## Graphical abstract

Fractionation and comprehensive characterization of C50 carotenoids from *Haloarcula rubripromontorii* BS2, identified all‐trans bacterioruberin as the predominant isomer. The haloarchaeal carotenoid exhibited strong antioxidant activity, photostability in oil and was biocompatible with human keratinocytes, highlighting their potential as sustainable natural antioxidants for dermatological applications.

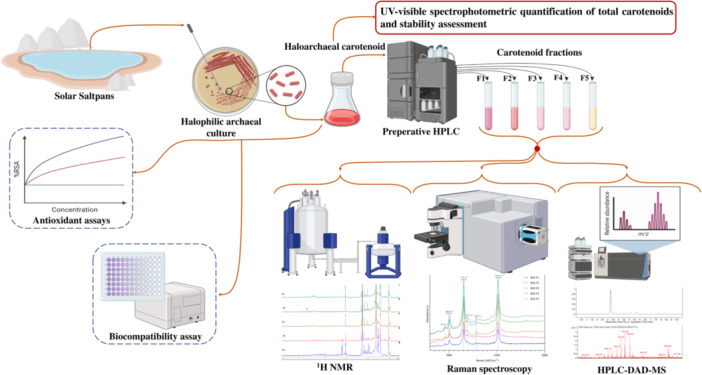

## 1 Introduction

Carotenoids belong to a category of naturally occurring chromophores biosynthesized by a diverse range of organisms, including archaea, bacteria, algae, plants, and some fungi (Sahli et al. 2021). The basic skeleton of carotenoids consists of the poly‐isoprenoid structure. They vary in the alkyl chain length, ranging between 30 and 50 carbons in length, and coloration ranging from yellow to red. Most abundant carotenoids are those having a C40 hydrocarbon backbone, while fewer contain 30, 35, 45 and 50 carbon atoms. Of these, the C45 and C50 carotenoids, referred to as ‘higher carotenoids’, have extended carbon backbones and often exhibit distinct structural and functional characteristics. One of the major potential applications of carotenoids is their antioxidant abilities which are largely dependent on their chemical composition, particularly degree of conjugation and alkyl chain length (Abbes et al. 2013; Jones and Baxter 2017; Grivard et al. 2022). Carotenoids are produced via terpenoid pathways, where the initial step involves biosynthesis of a C40 carotenoid, phytoene through the condensation of two molecules of geranylgeranyl pyrophosphate (GGPP). Phytoene then undergoes multiple desaturation reactions, leading to the formation of lycopene, which plays a crucial role in the C50 biosynthetic pathway for the production C50 carotenoids (Yang et al. 2015; Giani et al. 2020).

Haloarchaea are salt‐loving extremophiles that thrive in hypersaline environments such as brine lakes and solar evaporation ponds. They possess specialized metabolic mechanisms adapted to extreme environments, making them a valuable source of unique bioactive compounds with potential biological uses. The C50 carotenoids in haloarchaea are reported to provide greater lipophilicity and functional stability (Rodrigo‐Baños et al. 2015). They are crucial to haloarchaea by providing photo‐protection, aiding in pH adaptation, and maintaining their structural integrity. Additionally, they also scavenge free radicals like reactive hydroxyl species, singlet oxygen forms, and reactive nitrogen forms (Palanisamy and Ramalingam 2024).

Among the various intermediary metabolites and pigments, higher carotenoids, especially the C50 carotenoids remain underexplored, with minimal studies describing the molecular and biological characteristics of the same (Sahli et al. 2021). Given the growing need for novel biomolecules with different applications and the ease of extraction and purification of these molecules from extremophiles, we have explored the carotenoid composition from halophilic archaea. One of the major advantages of using haloarchaea for natural product extraction is they grow luxuriantly in high salt conditions thereby reducing the risk of microbial contamination even in non‐sterile environments. Lysis of the haloarchaeal cells with distilled water followed by well‐established purification protocols (i.e. aqueous/organic phase separation techniques) can yield high quantities of the desired product (Oren 2016; Torregrosa‐Crespo et al. 2017; Moopantakath et al. 2023). Recently, we have reported the genomic characteristics of *Haloarcula rubripromontorii* BS2, a strain found in the hypersaline environment of the salt pans of the western state of Goa in India (Nagar et al. 2024). This species produces a bright orange pigment and was identified to be the most dominant culturable member among the haloarchaea in hypersaline areas by our team (Mani et al. 2012). The present work is focused on extraction carotenoids from this haloarchaeon, their structural characterization, and analysing functional as well as biological properties. We found that these carotenoids exist in multiple isomeric forms of higher carotenoids with the C50 (all *trans*) being the major component. Since this isolate can be cultivated easily on both laboratory and industrial scales, and the costs associated with the isolation and purification of its carotenoids are economically feasible, this study paves the way for exploring *Haloarcula rubripromontorii* BS2 and its carotenoids for potential commercial applications. Furthermore, this work provides a comprehensive structural, functional, and biocompatibility analysis of C50 carotenoids extracted specifically from *Haloarcula* species, emphasizing their distinct antioxidant efficiency and significant promise for cosmeceutical development.

## 2 Material and Methods

### 2.1 Haloarchaeal Isolate and Culture Conditions

Halophilic archaeon *Haloarcula rubripromontorii* BS2 was isolated from Ribandar solar salt pans in Goa, India (Mani et al. 2012). The complete genome profile of BS2 strain has been submitted at GenBank with the accession number JAWJXX000000000 (Nagar et al. 2024). The isolate was deposited in the National Centre for Cell Science, Pune, India (MCC2588). The culture was maintained on extremely halophilic medium (EHM) composed of (gL^−1^) NaCl 250; MgSO_4_·7H_2_O 20.0; KCl 2.0; CaCl_2_·2H_2_O 0.36; NaHCO_3_ 0.06; NaBr 0.23; Peptone 5.0; yeast extract 10.0; FeCl_3_·6H_2_O 0.005 with the pH adjusted to 7.0–7.4 (Salgaonkar and Bragança 2015), at 37°C and 110 rpm in an orbital shaking incubator.

### 2.2 Growth Conditions of *Haloarcula rubripromontorii* BS2

*Haloarcula rubripromontorii* BS2 was grown in 500 mL EHM medium in 1 L Erlenmeyer flasks and inoculated with 2% (v/v) exponentially growing culture. The flasks were maintained under orbital shaking at 110 rpm and 37°C. The growth of the culture was spectrophotometrically recorded at 600 nm every 24 h for 14 days.

### 2.3 Extraction and Quantification of Total Carotenoids

Carotenoids were extracted after 10th day of incubation. Biomass was harvested by centrifugation at 10,000 × g for 20 min at 4°C and the sedimented cells were resuspended in 1 mL of acetone per 10 mL of culture broth. This was followed by a single freeze‐thaw cycle, where the cell pellet suspended in acetone was kept at −80°C for 1 h and then thawed at room temperature. Then it was sonicated over ice for 30 s pulses 3 times with 30 s breaks and the suspension was then vortexed and centrifuged at 10,000 × g to separate out the cell debris. The extraction process was continued until the cell pellet turned white in colour or colourless, and solvent containing bright orange carotenoid was collected. The extract was freeze‐dried and stored at −80°C covered in foil for further use. Carotenoid was quantified by the following formula:

$$C({\mathrm{µgmL}}^{-1})=\left({\text{OD}}_{\lambda \max}/\varepsilon_{1\text{cm}}^{1\%}\right)\times 10^{4}$$

The total carotenoid content (C) was determined by measuring the absorption maxima (*λ*_max_) and calculated using an extinction coefficient ε (1%) of 2540 for acetone (Britton et al. 2004; Montero‐Lobato et al. 2018; Sahli et al. 2021).

### 2.4 Analysis and Chromatographic Separation of Total Carotenoids

The carotenoid extract was analysed using UV‐visible double beam spectrophotometer (Shimadzu, Japan, UV‐2450) by scanning within the range of 300–600 nm.

The freeze‐dried carotenoids were re‐dissolved in acetone and separated on thin layer chromatography (TLC), Silica Gel 60 F254 sheet. The solvent system employed was a mixture of acetone and n‐heptane in 1:1 ratio, as described by Fang et al. (Fang et al. 2010). No specific visualization method was necessary, given that the compounds were coloured.

For HPLC analysis the freeze‐dried carotenoid extract was dissolved in methanol of HPLC‐grade and filtered via a 0.45 µm syringe filter to eliminate any particles. It was then analysed and fractionated using the preparative HPLC Shimadzu LabSolutions (Kyoto, Japan) equipped with a Photo array detector (SPD‐M40) and a binary pump (LC‐20AP). The column used was a Shim‐pack GIS C18 10 µm preparative column measuring 30 × 250 mm and the flow rate of 25 mL/min was maintained. The detection wavelength was set at 494 and 450 nm and 5 mL of sample volume was injected. The gradient mobile phase containing, A: methanol (MeOH) and B: Acetonitrile (ACN) in ratios as seen in Table 1 were used. The fractions were separately collected, pooled, lyophilized and stored for further characterization.

**Table 1:** Gradient schematic for separation of haloarchaeal carotenoid by preparative HPLC‐PDA.

Time (min)	A% (MeOH)	B % (ACN)
0.01	85	15
5.00	90	10
10.00	95	5
15.00	100	0
19.50	85	15
20.00	Stop	

### 2.5 Characterization of the Fractions

The fractions obtained from preparative HPLC were further characterized by a high‐performance liquid chromatograph (Agilent Technology, USA), equipped with a diode array detector (G7117A) coupled with a mass spectrometer and a quaternary pump (G7104A). This was analysed using a C18 POROSHELL analytical column (2.6 µm, 4.6 × 50 mm) and the sample injection volume of 7 µL was set. The mobile phase was composed of 90% methanol, 5% acetonitrile and 5% sterile MilliQ water containing 0.1% formic acid, with a flow rate of 0.5 mL/min. To reduce the oxidation and isomerization of the carotenoid, samples were kept on ice and wrapped with aluminium foil. The temperature was maintained at 25°C and UV detection was performed at 450 and 494 nm.

The Mass spectra within the range of 500 to 900 m/z, including [M + H]^+^, [M+Na]^+^, and [M + K]^+^ ions, were obtained using the 6460 Triple Quadrupole LC/MS system featuring electrospray ionization source (ESI) in positive ion mode. Working parameters included nebulizer pressure of 35 psi, drying gas flow of 10 L/min, gas temperature of 350°C and capillary voltage of 4000 V. Carotenoids and its isomers were identified via data analysis of the UV‐visible spectra, spectral fine structure and characteristics of the mass spectra.

Vibrational spectra of the fractions were recorded on Raman spectroscopy LAB RAM HR Horiba (France), at excitation wavelength 532 nm (spectral range of 800–1700 cm− 1) and acquisition time of 10 s.

For the nuclear magnetic resonance (NMR) spectroscopic analysis the freeze‐dried carotenoid fractions were dissolved in DMSO‐d6 (deuterated DMSO) and *trans*ferred to 5 mm NMR tubes. ^1^H NMR spectrum was acquired using a Bruker NMR spectrometer 500 MHz equipped with Avance neo console and the chemical shifts were reported in ppm (δ). Data was acquired and processed with Topspin 4.2 software (Bruker BioSpin GmbH, Germany).

### 2.6 Stability of Haloarchaeal Carotenoids

The carotenoids were dissolved in acetone and olive oil and exposed to intense sunlight (between 11 AM and 1 PM) for 30 min. Spectral scans were obtained from 300–600 nm before and after exposure to sunlight.

### 2.7 Evaluation of the Antioxidant Property

The antioxidant activity of the total carotenoids was quantified by the 2,2‐Diphenyl−1‐picrylhydrazyl (DPPH) radical scavenging assay as per Brahma et al. (Brahma and Dutta 2022) with some modifications. Briefly, 50 µL of the sample ranging from 1.5 to 30 µgml^−1^ was mixed with 100 µL of 0.25 mM DPPH solution in methanol and incubated for 30 min in dark. Fifty microliters of methanol in 100 µL of DPPH was considered as control. Optical density was recorded at a wavelength of 517 nm. The antioxidant assay was performed in triplicates, and the radical scavenging activity (RSA) of the samples was estimated through the formula below: (Chintong et al. 2019)

$$\text{DPPH}\;\text{radical}\;\text{scavenging}\;\text{ability}\left(\%\right)=\left[A_{C}-\left(A_{S}-A_{\text{SB}}\right)/A_{C}\right]\times 100$$

where, A_C_, A_S_ and A_SB_ refer to the absorbances of the DPPH solution (control), carotenoids with DPPH (test sample) and carotenoids without DPPH (sample blank) respectively.

Antioxidant activity was also measured by 2,2'‐azino‐bis(3 ethylbenzothiazoline‐6‐sulfonic acid (ABTS) radical scavenging assay carried out as per Patkar et al. (Patkar et al. 2021) with some modifications. Briefly, 7 mM of ABTS solution was reacted with 2 mM of potassium persulphate and the mixture was kept in the dark for 12–16 h at room temperature. The resulting ABTS final solution was diluted with water to a stable absorbance of 0.70 ± 0.05 at wavelength of 734 nm. In 96‐well plates, 50 µL of the sample ranging from 1.5 to 30 µgmL^−1^ was mixed with 100 µL of ABTS solution and incubated in the dark for 6 min. A control of 50 µL of methanol in 100 µL of ABTS was kept. Absorbance was recorded at 734 nm. The antioxidant assay was performed in triplicates, and the radical scavenging activity (RSA) of the samples was estimated through the formula below: (Chintong et al. 2019)

$$\text{ABTS}\;\text{radical}\;\text{scavenging}\;\text{ability}\left(\%\right)=\left[A_{C}-\left(A_{S}-A_{\text{SB}}\right)/A_{C}\right]\times 100$$

where, A_C_, A_S_ and A_SB_ refer to the absorbance of the ABTS solution (control), carotenoids with ABTS (test sample) and carotenoids without ABTS (sample blank) respectively.

### 2.8 Effect of Haloarchaeal Carotenoids on Skin Cell Line

#### 2.8.1 Cell Culture and Morphology

Human keratinocyte cell line (HaCaT) was regularly cultured in Dulbecco's modified Eagle's medium (DMEM) supplemented with 10% fetal Bovine serum (FBS), henceforth referred to as complete medium, at 37°C in a humidified incubator with 5% CO_2_. All cell culture reagents were obtained from Himedia. The cells were passaged once they attained 80%–90% confluence and assays were conducted when they attained 70%–75% confluence. The cells were treated with the carotenoids for 24 h.

#### 2.8.2 Cell Viability Assay

The cell viability was evaluated using 3‐(4,5‐dimethylthiazol‐2‐yl)‐2,5‐diphenyltetrazolium bromide (MTT) assay. Briefly, 1 × 10^4^ HaCaT cells were seeded per well in 96‐well plates. After confluency, the medium was replaced with different concentrations (1.5, 2.5, 5, 10, 20 and 50 µgmL^−1^) of carotenoids dissolved in DMEM with DMSO (the final concentration of DMSO was maintained less than or equal to 0.1%) and incubated for 24 h. Thereafter, it was replaced with MTT solution for 4 h. After incubation the formazan crystals formed were solubilized in 150 µL of DMSO, and the viability was quantified at 570 nm (with a reference at 630 nm) using a microplate reader. The experiments were performed in triplicates.

### 2.9 Statistical Analyses

Data was analysed using GraphPad Prism (version 9.5.0). The results were expressed as mean ± SD and the IC_50_ values were obtained from the non‐linear regression plots. One‐way ANOVA was performed to analyse difference between means with *p* < 0.05 level of significance.

## 3 Results and Discussion

### 3.1 Growth and Total Carotenoid Quantification

**Figure 1:**
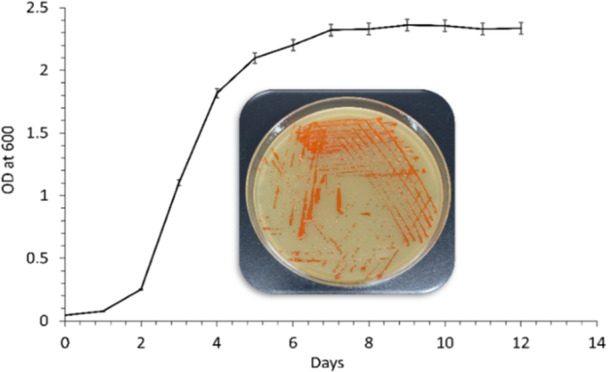
Growth curve of *Haloarcula rubripromontorii* BS2 in EHM broth and on the EHM agar plate (inset).

Growth of *Haloarcula rubripromontorii* BS2 (GenBank accession number JAWJXX000000000) was recorded for 14 days (Figure 1). The culture attained a bright orange coloured pigment from the 5th day onwards. The carotenoid was extracted in acetone on the 10th day of growth and showed a yield of 11.74 ± 1.02 µgmL^−1^.

### 3.2 Analysis and Chromatographic Separation of Total Carotenoids

**Figure 2:**
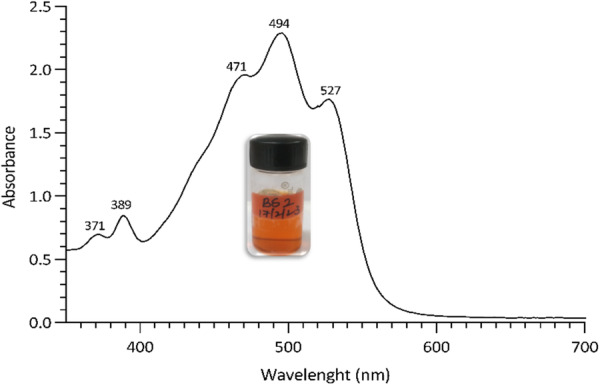
UV–Visible spectra of pigment extracted from *Haloarcula rubripromontorii* BS2. Inset shows the pigment extract in acetone.

Carotenoids extracted from *Haloarcula rubripromontorii* BS2 displayed an absorption profile with characteristic three‐fingered peaks at 468, 494, and 528 nm and distinctive minor *cis* signals at 370 and 388 nm (Figure 2). The absorption spectra of these carotenoids closely resembled the previous reports on red pigmented C50 carotenoids, which had approximate absorption maxima around 467, 493, and 527 nm along with two *cis* peaks at 370 and 385 nm (Abbes et al. 2013; Shahbazi et al. 2023). One of the primary criteria for identifying and characterizing carotenoids is its light‐absorbing ability. These carotenoids have a long‐conjugated double‐bond system (the chromophore) attributing intense light absorption prominently in the visible region around the 450–550 nm range or, in some cases in the UV regions (Britton et al. 2004; Calegari‐Santos et al. 2016). It is important to understand that the UV‐visible spectrum provides information only about the chromophore of the molecule and has little to no information on the functional groups (Britton et al. 2004). The carotenoid extract resolved into four reddish‐orange spots on TLC, with spot 1 exhibiting the most intense colouration (Supplementary Fig. 1), with the Rf values of 0.39 (spot 1), 0.47 (spot 2), 0.50 (spot 3), and 0.55 (spot 4). These values align with the earlier reports of *Haloferax* sp., pigments (Ronnekleiv 1995; Strand et al. 1997; Fang et al. 2010).

**Figure 3:**
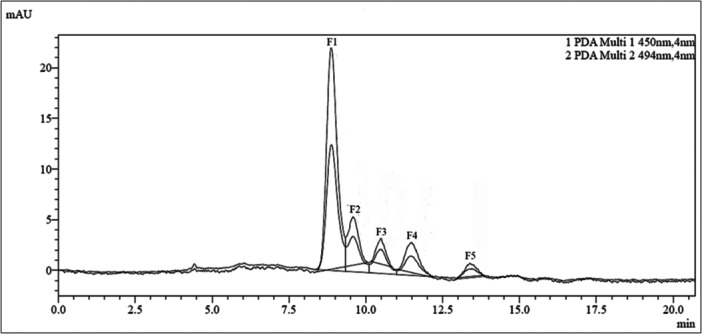
Preparative HPLC profile of the carotenoids from *Haloarcula rubripromontorii* BS2.

Fractionation of the haloarchaeal carotenoids by preparative HPLC resulted in five distinct fractions (Figure 3). The most abundant (61.6%) of these fractions, fraction 1 (F1), had a retention time of 8.9 min. Fractions 2–5 (F2‐F5) had retention times of 9.6, 10.5, 11.5, and 13.4 min with an abundance of 14.4%, 7.9%, 11.7%, and 4.6% respectively.

### 3.3 Characterization of the Separated Carotenoid Fractions

Detailed characterization of each fraction using LC‐MS, NMR and Raman spectroscopy is as follows:

Structural analysis of fraction 1:

The first fraction, when analysed by LC‐MS resolved into five subfractions. The first subfraction (F1‐1), eluted at 3.222 min and was characterized as all‐*trans*‐bacterioruberin (all‐*trans*‐BR). This subfraction represented the most prominent peak, constituting 80.3% of the total carotenoid in the entire first fraction. The rest of the subfractions (F1‐2, 3, 4 and 5) had retention times of 3.618, 4.419, 5.112 and 5.679 min, respectively. They were identified as *cis* geometrical isomers of bacterioruberin, accounting for 2.2%, 1.7%, 7% and 8.9% of the total area respectively. These are reported in Table 2 with the [M + Na]^+^ ion at m/z of 764.2 and an adjacent peak at [M + H]^+^ ion at *m*/*z* of 742.2. The mass spectra of these *cis* isomers resemble all‐*trans*‐bacterioruberin. However, their UV–visible spectra, which displayed hypsochromic shifts, showed less fine structure and a distinct *cis* peak than the *trans* isomer (Supporting Information Figure 3). Differentiation between these isomers became evident as the intensity of the *cis* peak increased as the *cis* double bond got closer to the centre of the molecule (Mandelli et al. 2012).

**Table 2:** Characteristics of the fractions obtained from *Haloarcula rubripromontorii* BS2 carotenoid determined using HPLC‐DAD‐MS.

Fraction	Subfraction	RT (min)	*Cis λ* max (nm)	λ max (nm)	$\%A_{\mathrm{III}}/A_{\mathrm{II}}$	$\%A_{\mathrm{cis}}/A_{\mathrm{II}}$	Molecular weight	Molecular Ion [M + H] + /[M + Na]^+^ (m/z)	Tentative identification
Fraction: 1 (F1)	F1‐1	3.222	371, 388	468, 495, 528	87.3	12.1	740.6	764.2	all‐*trans*‐BR
	F1‐2	3.618	368, 385	461, 487, 519	80.8	23.4	740.6	764.2	5‐*cis*,9’‐*cis*‐BR
	F1‐3	4.419	370, 386	467, 492, 525	83.8	18.9	740.6	764.2	5‐*cis*‐BR
	F1‐4	5.112	370, 387	462, 489, 521	86.1	27.8	740.6	764.2	9‐*cis*‐BR
	F1‐5	5.679	370, 386	461, 489, 521	80	81.7	740.6	764.2	13‐*cis*‐BR
Fraction: 2 (F2)	F2‐1	3.420	371, 388	468, 495, 528	82.8	15.5	722.5	723.1	all‐*trans*‐MABR
	F2‐2	3.843	367, 384	460, 484, 516	80.8	25.1	722.5	723.2	9‐*cis*‐MABR
	F2‐3	4.050	367, 384	459, 486, 517	79	64.3	722.5	723.2	13‐*cis*‐MABR
	F2‐4	4.707	369, 386	465, 492, 525	87	12.7	722.5	723.2	5‐*cis*‐MABR
Fraction: 3 (F3)	F3‐1	3.492	370, 388	473, 494, 527	81	17.9	740.6	764.2	BR
	F3‐2	5.472	370, 387	461, 489, 521	84.7	27.1	740.6	764.3	9‐*cis*‐BR
	F3‐3	6.066	370, 386	461, 489, 521	78.9	80.1	740.6	764.2	13‐*cis*‐BR
Fraction: 4 (F4)	F4‐1	3.465	371, 388	468, 495, 528	86.9	13.7		804.3	ND
	F4‐2	5.499	370, 387	462, 489, 521	83.4	29.1	740.6	764.2	9‐*cis*‐BR
	F4‐3	6.075	370, 386	462, 489, 521	79.3	74.8	740.6	764.2	13‐*cis*‐BR
Fraction: 5 (F5)	F5‐1	7.425	370, 387	468, 494, 528	83.8	17.3	722.5	724.2	5‐*cis*‐MABR
	F5‐2	8.235	368, 385	461, 487, 519	81.8	25.6	722.5	723.2	9‐*cis*‐MABR

Abbreviations: RT, Retention time of the subfractions; $\%A_{\mathrm{III}}/A_{\mathrm{II}}$, spectral fine structure, $\%A_{\mathrm{cis}}/A_{\mathrm{II}}$, *cis* peak intensities. Bacterioruberin (BR), Monoanhydrobacterioruberin (MABR).

**Figure 4:**
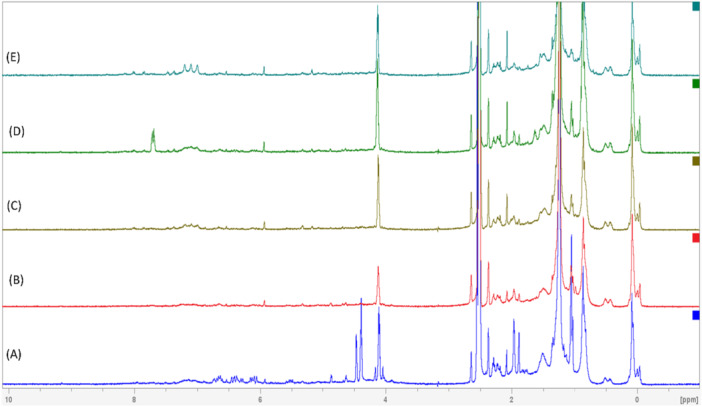
NMR spectra of the fractions obtained from *Haloarcula rubripromontorii* BS2 carotenoid (A) F1, (B) F2, (C) F3, (D) F4, (E) F5.

**Figure 5:**
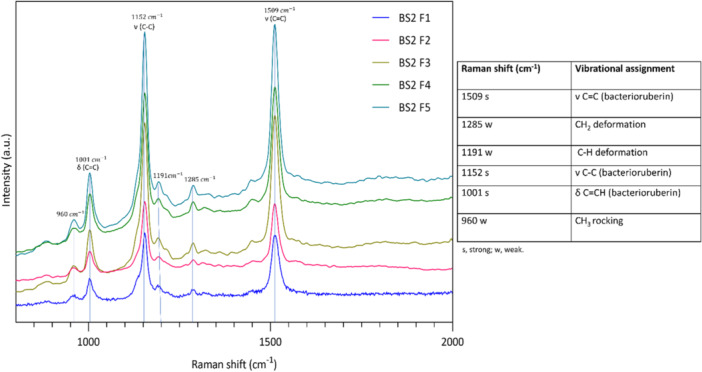
Raman spectra of the fractions obtained from *Haloarcula rubripromontorii* BS2 carotenoid. Their typical vibrational peak assignments and intensities observed by Raman spectroscopy are detailed.

NMR spectroscopy was used to get finer structural details of the different fractions. The conjugated olefinic protons (C = C hydrogens) in bacterioruberin are usually downfield shifted and are observed in the chemical shift range of 6–7 ppm. The hydroxyl protons (the alcohol protons) were observed in the 4–5 ppm range, while the terminal methyl protons were upfield shifted and resonated at 0.8–1.5 ppm. The branched methyl protons were slightly downfield shifted and resonated at 1.2–2.2 ppm (Figure 5). Though fraction 1 consisted of five different isomers of bacterioruberin (BR) as observed from LC‐MS data, the predominant variant is the all‐*trans* BR, and the NMR spectrum is also indicative of this observation, with peaks corresponding to the chemical shifts mentioned above (Figure 4A).

The Raman spectroscopic analysis was also employed to elucidate the structural details of the carotenoids in all five fractions. All the haloarchaeal carotenoid fractions showed vibrational spectral bands at 1509 and 1152 cm^−1^ corresponding to C = C and C–C stretching vibrations indicating the polyene chain of C50 carotenoids (bacterioruberin). The bands at 1001 cm^−1^ corresponding to C = CH also indicated C50 carotenoids. Bacterioruberin has a molecular configuration of conjugated isoprenoid system with 13 C = C units and four ‐OH or hydroxyl functional groups with no subsidiary conjugation arising from the terminal groups. The weaker band at 1191 cm^−1^ corresponding to C‐H deformation vibrations in conjugated systems, potentially coupled with stretching vibrations of the conjugated C‐C bonds. Additionally, weaker bands at 1285 and 960 cm^−1^ corresponding to CH_2_ deformation vibrations and CH_3_ rocking respectively were also identified. Thus, Raman vibration spectra of all the fractions showed the same spectral patterns (Figure 5) indicating the presence of all‐*trans* bacterioruberin and its possible chemical analogues in all the collected fractions (Marshall et al. 2006, 2007; Jehlička et al. 2013).

Structural analysis of fraction 2:

The second fraction resolved into four subfractions, with the 4th subfraction (F2‐4) having a retention time of 4.707 min and was identified as an all‐*trans*‐monoanhydrobacterioruberin (all‐*trans*‐MABR) and displaying the highest relative abundance, accounting for 75.61%. Whereas subfraction peaks from 1 to 3 (F2‐1, 2 and 3) had retention times of 3.420, 3.843 and 4.050 min respectively constituting of 12.7%, 5.39% and 6.3% of the total peak area, respectively. These subfraction peaks correspond to various *cis* isomers of monoanhydrobacterioruberin (MABR). All these isomers had similar mass spectra with [M + H]^+^ ion at m/z of 723.1‐723.2, with varying UV‐visible spectra (Supplementary Fig. 4). This data agrees with previous reports on all‐*trans*‐MABR being abundant among the MABR isomers (Ma et al. 2023). MABR is one of the metabolic intermediate compounds during the synthesis of bacterioruberin (Nagar et al. 2024).

The ^1^H 1D NMR spectrum of this fraction showed the characteristic peaks of BR (Figure 4B); nevertheless, the intensities of the peaks were slightly less than those of fraction 1, indicating the differences in the concentrations of these carotenoids in the collected fractions.

The Raman spectrum of fraction 2 showed similar vibrational spectral bands to those of the first fraction (Figure 5), with variations in peak intensities that could be attributed to differences in the concentrations of the collected fractions.

Structural analysis of fraction 3:

The third fraction resolved into three subfractions. The first subfraction (F3‐1) constituted 60.37% of the total fraction in the third fraction, thus being the most abundant isomer with a retention time of 3.492 min. This was followed by second and third peaks (F3‐2 and 3) accounting for 33.54% and 6.09% of the total area with retention times of 5.472 and 6.066 min, respectively. Each of these subfractions corresponded to majorly *cis* isomers of bacterioruberin (BR) with [M + H]^+^ ion at *m*/*z* of 742.2 and adjacent peak at [M + Na]^+^ ion at m/z of 764.2. The UV‐visible spectrum had a lower fine structure and higher *cis* peak intensity compared to all‐*trans* isomers (Supporting Information Figure 5) (Mandelli et al. 2012).

^1^H 1D NMR spectrum and Raman spectrum of this fraction displayed the characteristic peaks associated with BR (Figure 4C); however, the peak intensities were slightly varied than other fractions, suggesting variations in the concentrations of these carotenoids within the collected fractions.

Structural analysis of fraction 4:

The fourth fraction resolved into three subfractions, containing unidentified carotenoid and isomers of bacterioruberin. The first subfraction (F4‐1) had an m/z value of 804.3. The three‐fingered UV‐visible absorption patterns of this particular peak indicated it to be a form of a carotenoid. Unambiguous identification of the type of carotenoid couldn't be carried out due to the very low resolution of the MS data (Supporting Information Figure 6). This subfraction was the most abundant peak with 83.86% total carotenoid in the 4th fraction, with a retention time of 3.465 min. Interestingly, an additional peak at 7.8 ppm was seen in this fraction other than characteristic peaks associated with BR for the ^1^H 1D NMR spectrum (Figure 4D). However, further analysis needs to be carried out using 2D NMR studies to probe the nature of the variants present in this fraction. The second and third subfractions (F4‐2 and 3) with retention times of 5.499 and 6.075 min respectively corresponded to bacterioruberin (BR) with [M + H]^+^ ion at *m*/*z* of 742.2 and adjacent peak at [M + Na]^+^ ion at m/z of 764.2. These subfractions contributed minimally to the total carotenoid in the fourth fraction, accounting for 6.8% and 9.34%, respectively.

Structural analysis of fraction 5:

The fifth fraction had two subfractions, F5‐1 and F5‐2 with retention times of 7.425 and 8.235 min respectively. F5‐1 and F5‐2 constituted 95.14% and 4.86% of the total peak area respectively and corresponded to *cis* isomers of monoanhydrobacterioruberin (MABR) with [M + H]^+^ ion at m/z of 724.2 and 723.2 respectively (Supporting Information Figure 7).

Different mass spectra contributed to the identification of bacterioruberin and its intermediate compound and could not be used to distinguish between the isomers. Therefore, the structural assignments were examined by their UV‐ visible spectra. Each subfraction was analysed corresponding to their maximum wavelengths, from which the spectral fine pattern (%AIII/AII) and the *cis* peak intensity (%A*cis*/AII) were calculated (Supporting Information Figure 3–7). All the subfraction peaks, displayed UV‐visible absorption spectra with photometric characteristics typical of bacterioruberin‐like carotenoids, and significant differences in absorbance intensities were identified at their UV and visible absorption maxima. The absorbance maxima at 484–494 nm (peak II), 516–528 nm (peak III), and 386–388 nm (*cis*‐peak) are mentioned in Table 2 (Flores et al. 2020; Serino et al. 2023; Shahbazi et al. 2023).

Fraction 5 exhibited two isomeric forms of monoanhydrobacterioruberin (MABR) 5‐*cis*‐MABR and 9‐*cis*‐MABR, with 5‐cis‐MABR being the most abundant. ^1^H 1D NMR spectrum of fraction 5 displayed similar characteristic peaks associated with BR (Figure 4E), with few variations in the intensities of the peak.

*Haloarcula rubripromontorii* BS2 exhibited a distinctive carotenoid profile, wherein interestingly bisanhydrobacterioruberin (BABR), a biosynthetic intermediate was not detected, but was found in previously studied *Haloarcula* species. *Haloarcula japonica* was reported to have a carotenoid composition consisting of bacterioruberin (BR, 68.1%), monoanhydrobacterioruberin (MABR, 22.5%), and bisanhydrobacterioruberin (BABR, 9.3%) (Yatsunami et al. 2014). In contrast, *Har. rubripromontorii* BS2 lacked BABR while maintaining elevated levels of BR (69.5%) and MABR (19%). BABR serves as a biosynthetic precursor that undergoes sequential enzymatic conversion to MABR via CruF hydratase activity and MABR subsequently transformed to the final product BR. Previous reports on carotenoids from various *Haloarcula* species and other halophilic archaea, such as *Halobacterium salinarum*, *Haloferax volcanii* and *Halorubrum* sp. have detected BABR (Yang et al. 2015; Ma et al. 2023, 2024; Rodrigo‐Baños et al. 2015). The absence of this intermediate in *Har. rubripromontorii* BS2 suggests exceptionally efficient metabolic conversion, where BABR is rapidly processed without detectable accumulation. Moreover, detailed characterization the carotenoids in *Har. rubripromontorii* BS2 helped identify the abundance of all‐trans configurations along with some cis isomers. This is a comprehensive study of *Har. rubripromontorii* BS2 carotenoids for advancing our understanding of C50 carotenoid biosynthesis mechanisms in halophilic archaea.

### 3.4 *Haloarcula rubripromontorii* BS2 Carotenoids Are Most Stable in Oil

The stability of the haloarchaeal carotenoids was analysed in solvents such as acetone and olive oil. The carotenoid in acetone became colourless after 30 min of exposure to intense sunlight with decline in the UV‐visible peaks as the exposure time progressed. A notable reduction in the absorbance intensity was recorded every 5 min (Supporting Information Figure 2A). Whereas olive oil prevented degradation of the haloarchaeal carotenoids, and the colour persisted even after half an hour of exposure to intense sunlight. The peak intensity displayed negligible fluctuation even after 30 min of exposure to sunlight (Supporting Information Figure 2B). Carotenoid synthesis by haloarchaea is known to fluctuate with variations in salinity, dissolved oxygen, etc. and is also affected by light and heat (Fang et al. 2010; Rodrigo‐Baños et al. 2015; Calegari‐Santos et al. 2016). When assessing the stability of the carotenoids in polar solvents, it was observed that the haloarchaeal carotenoid decolorized upon exposure to sunlight. C50 carotenoids are integrated into the lipid bilayer which protects them from oxidation. However, solvents like methanol or acetone cannot provide this protective environment, thus making it susceptible to photodegradation. This leads to the conversion of the *trans*‐isomeric form of carotenoids to *cis*, which is less stable and thereafter undergoes further breakdown resulting in loss of colour and antioxidant activities, while oils provide better stability and mimic the natural lipid environment. This is one of the main reasons that the extraction and storage of carotenoids is done in the dark, especially when polar solvents are involved (Miękus et al. 2019). Here, we observed that the use of oils could markedly maintain the stability of the carotenoids as compared with solvents.

### 3.5 *Haloarcula rubripromontorii* BS2 Carotenoids Display Significant Antioxidant Activity

**Figure 6:**
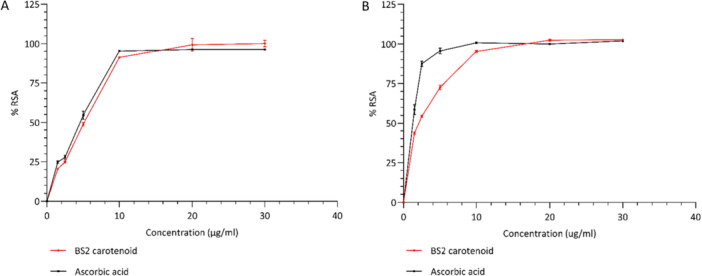
The percent radical scavenging activity (%RSA) of carotenoid from *Haloarcula rubripromontorii* BS2 at different concentrations evaluated by DPPH (A) and ABTS (B). Each % RSA value is the mean ± standard deviation of three replicate analyses.

The radical scavenging percentage calculated by both the assays, DPPH (Figure 6A) and ABTS (Figure 6B) showed an increase in %RSA in a concentration‐dependent manner, with an increase in the carotenoid content. The carotenoid extract showed IC_50_ values of 4.31 ± 0.07 and 2.04 ± 0.02 µgmL^−1^ for DPPH and ABTS respectively. Ascorbic acid (vitamin C) was taken as positive control and had IC_50_ values of 3.83 ± 0.08 and 1.32 ± 0.1 µgmL^−1^ for DPPH and ABTS respectively. The carotenoid from *Haloarcula rubripromontorii* BS2 showed strong radical scavenging activity, which is almost equivalent to ascorbic acid (Figure 6). This antioxidant properties of carotenoid from *Haloarcula rubripromontorii* BS2 are ascribed to its chemical structure as mostly antioxidant properties depend on the number of conjugated π‐bond system, their length and ‐OH groups. Thus, carotenoids that possess a greater length with maximum overlaps of conjugate double bond demonstrate stronger antioxidant activity (Mandelli et al. 2012; Ramesh et al. 2019). As bacterioruberin (mainly all‐*trans* isomer) is the major carotenoid, and possesses a longer hydrocarbon chain with 13 conjugated π‐bonds along with four terminal hydroxyl (‐OH) functional groups, which contribute to its remarkable scavenging activity. On the other hand, C40 carotenoids like β‐carotene a well‐known antioxidant comprises of only nine conjugated double bonds (Rodrigo‐Baños et al. 2015; Giani et al. 2019; Reis‐Mansur et al. 2019; Grivard et al. 2022). There have been only a few studies reporting strong antioxidant activity of the C50 carotenoids in *Haloarcula* species, and our findings with *Haloarcula rubripromontorii* BS2 add important evidence to this area. In the present study, the *Har. rubripromontorii* BS2 carotenoid extract demonstrated strong free radical scavenging activity (RSA), with IC₅₀ values of 4.31 ± 0.07 µgmL^−1^ (DPPH) and 2.04 ± 0.02 µgmL^−1^ (ABTS), which was matching or even exceeding the previously reported *Haloarcula* species. Specifically, *Har. rubripromontorii* BS2 displayed comparable activity to *Haloarcula hispanica* HM1 (DPPH = 2.06 µgmL^−1^; ABTS = 3.89 µgmL^−1^) (Gómez‐Villegas et al. 2020). While it outperformed several *Haloarcula* strains isolated from Atacama Desert, including TeSe‐41 (IC₅₀: 6.25 and 9.32 µgmL^−1^), ALT‐23 (8.83 and 12.12 µgmL^−1^), TeSe‐51 (15.43 and 18.45 µgmL^−1^), and TeSe‐89 (23.19 and 34.72 µgmL^−1^) (Lizama et al. 2021). These findings highlight the exceptional antioxidant potential of carotenoid from *Har. rubripromontorii* BS2, thus establishing it as one among the potent radical scavenging compounds reported.

### 3.6 *Haloarcula rubripromontorii* BS2 Carotenoids Are Biocompatible

**Figure 7:**
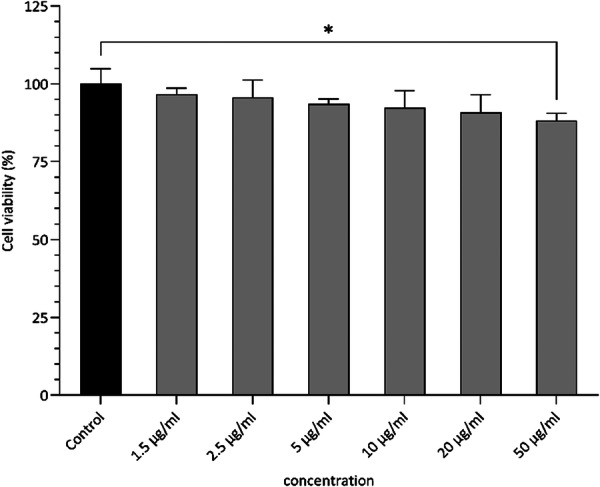
Evaluation of *Haloarcula rubripromontorii* BS2 carotenoids on cell viability of HaCaT keratinocytes. Cells were treated with increasing concentrations of the carotenoid extracts for 24 h. and the viability percentage was measured using MTT assay. The values represent the mean ± SD of three repetitions. *p < 0.05 indicates significant differences when compared to the control group (black bars).

Human skin keratinocyte (HaCaT) cells upon treatment with varying dosages of haloarchaeal carotenoids from 1.5 to 50 µgmL^−1^ concentrations, adhered and proliferated to form a confluent monolayer even at maximum dosage levels of 50 μg/mL and around 88% of cell viability remained viable (Figure 7). This aligns well with other research groups studying haloarchaeal carotenoids. For instance, Giani et al. (2023) demonstrated that bacterioruberin‐rich carotenoid extracts (BRCE) from *Haloferax mediterranei* selectively affected cancer cell lines while showing minimal toxicity to normal mammary epithelial cells (184A1), which maintained normal morphology and viability even at higher concentrations. Similarly, (Baeza‐Morales et al. 2025) demonstrated that carotenoid extract from *Haloferax mediterranei* was well‐tolerated by healthy peripheral blood immune cells, supporting the compatibility across diverse cell types. Additionally, it was seen that bacterioruberin from *Haloferax marinum* was well tolerated by C2C12 myotubes (Lee et al. 2024).

Together, these studies create a consistent output that haloarchaeal C50 carotenoids are biocompatible compounds at physiologically relevant concentrations. This indicates that carotenoids from *Haloarcula rubripromontorii* BS2 fit seamlessly with the literature, are not cytotoxic to normal cell lines and are safe for further applications in cosmeceuticals and other biological applications.

## 4 Conclusions

The strong antioxidant capacity and photostability in oils, combined with excellent skin keratinocyte (HaCaT) cell compatibility, indicate that these carotenoids can serve as natural bioactives in cosmeceutical formulations, particularly for anti‐aging and skin‐protective applications. Their safety and efficacy profile supports their translational relevance in the development of sustainable, haloarchaea derived skincare products.

## Author Contributions

**Devika N. Nagar:** writing – original draft, methodology, investigation, formal analysis, data curation. **Deepthi Das:** methodology, investigation. **Raviprasad Aduri:** investigation, data curation, writing – review and editing. **Judith M. Braganca:** investigation, writing – review and editing, project administration, funding acquisition. All authors read and approved the final manuscript.

## Ethics Statement

The authors have nothing to report.

## Conflicts of Interest

The authors declare no conflicts of interest.

## Supporting information


**Supporting Figure 1:** TLC profile of the carotenoid extract of *Haloarcula rubripromontorii* BS2. **Supporting Figure 2:** UV‐visible spectra of the carotenoids from *Haloarcula rubripromontorii* BS2. **Supporting Figure 3:** Chromatographic, Mass and Spectrometric analysis of subfractions (F1‐1 to 5) from fraction 1 (F1) of the carotenoid extract of *Haloarcula rubripromontorii* BS2. **Supporting Figure 4:** Chromatographic, Mass and Spectrometric analysis of subfractions (F2‐1 to 4) from fraction 2 (F2) of the carotenoid extract of *Haloarcula rubripromontorii* BS2. **Supporting Figure 5:** Chromatographic, Mass and Spectrometric analysis of subfractions (F3‐1 to 3 of fraction 3 (F3) from the carotenoid extract of *Haloarcula rubripromontorii* BS2. **Supporting Figure 6:** Chromatographic, Mass and Spectrometric analysis of subfractions (F4‐1 to 3) from fraction 4 (F4) of the carotenoid extract of *Haloarcula rubripromontorii* BS2. **Supporting Figure 7:** Chromatographic, Mass and Spectrometric analysis of subfractions (F5‐1 and 2) from fraction 5 (F5) of the carotenoid extract of *Haloarcula rubripromontorii* BS2.

## Data Availability

The data supporting this article have been included as part of the ESI.
