# Supplementary material for: Characterization and Functional Evaluation of Carotenoids From Haloarcula rubripromontorii BS2
Source: Microbiologyopen. 2026 Feb 5;15(1):e70228. doi: 10.1002/mbo3.70228 (PMC12877311; doi:10.1002/mbo3.70228)

Characterization and functional evaluation of the carotenoids from *Haloarcula rubripromontorii* BS2.

Devika N. Nagar^1^, Deepthi Das^1^, Raviprasad Aduri^1,2^ and Judith Maria Braganca^1^*

^1^Department of Biological Sciences, Birla Institute of Technology and Science, Pilani, K K Birla Goa Campus, NH 17 B, Zuarinagar, Goa, India. 403726

2Department of Chemistry, Birla Institute of Technology and Science, Pilani, K K Birla Goa Campus, NH 17 B, Zuarinagar, Goa, India. 403726


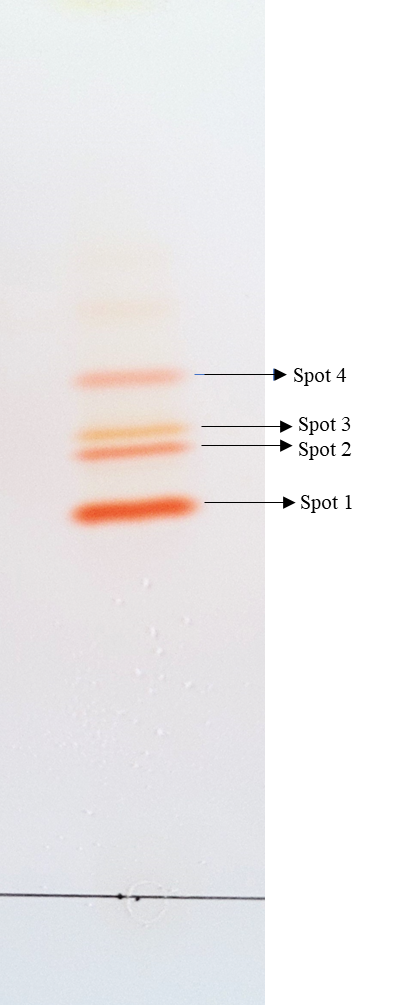


**Supplementary Fig 1**: TLC profile of the carotenoid extract of Haloarcula rubripromontorii BS2.


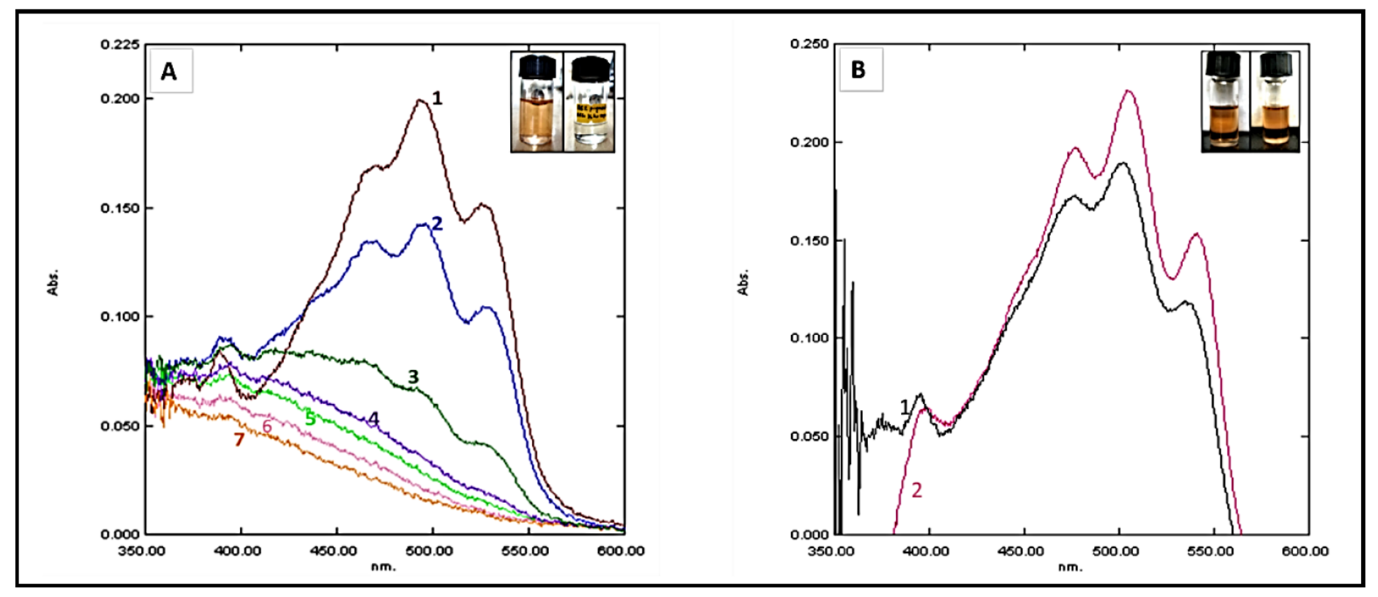


**Supplementary Fig 2**: UV-visible spectra of the carotenoids from Haloarcula rubripromontorii BS2; (A) Carotenoid dissolved in acetone and exposed to bright sunlight. Scanning done every 5-minute interval. 1- unexposed pigment, 2- 5 min exposure, 3- 10 min exposure, 4- 15 min exposure, 5- 20 min exposure, 6-25 min exposure, 7- 30 min exposure (B) Carotenoid dissolved in olive oil and exposed to bright sunlight. Scanning done after 0 and 30 minutes of exposure. 1- unexposed carotenoid, 2-30min exposure. Inset shows the carotenoid extract before and after 30 minutes’ exposure to sunlight.


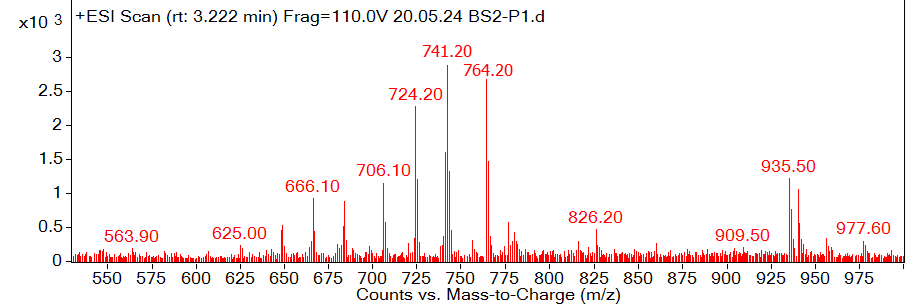

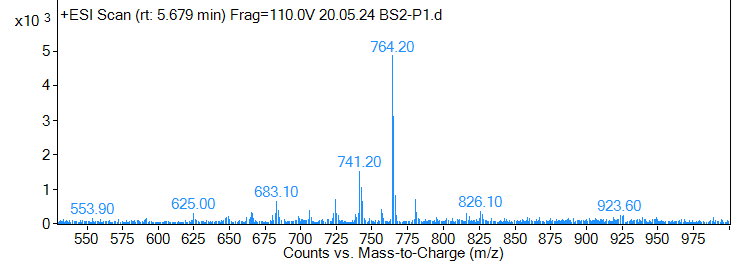

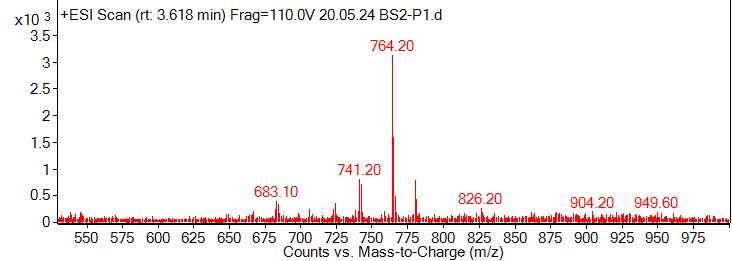

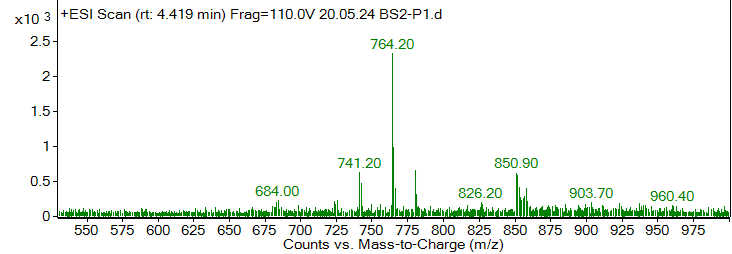

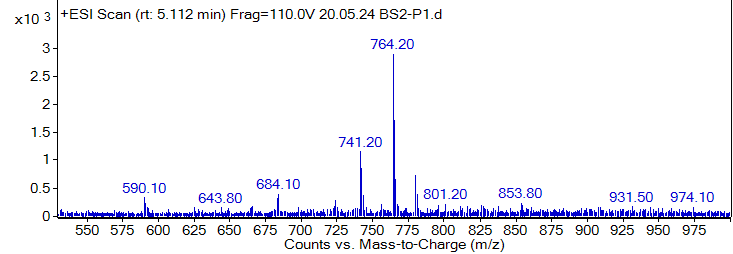

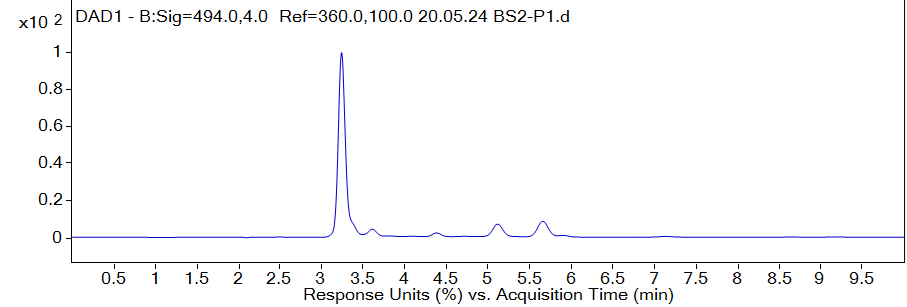

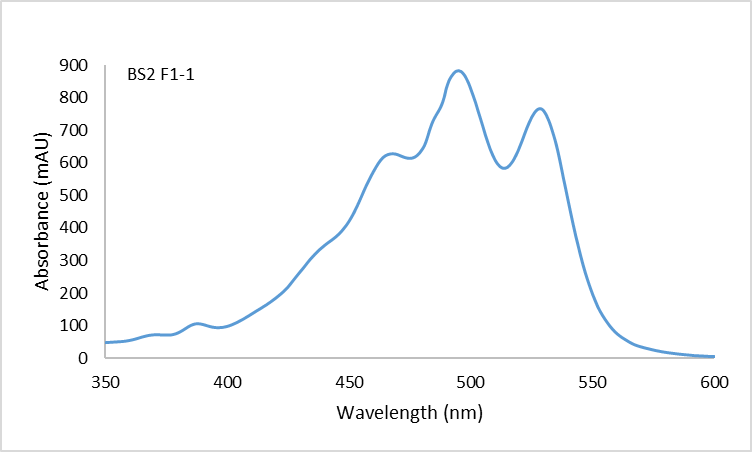

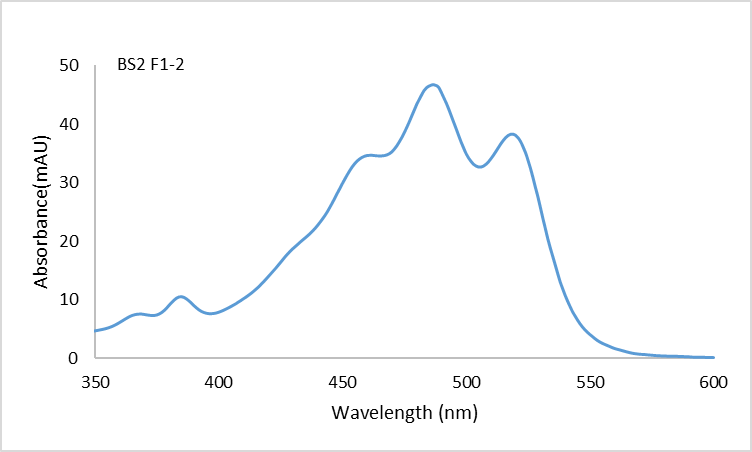

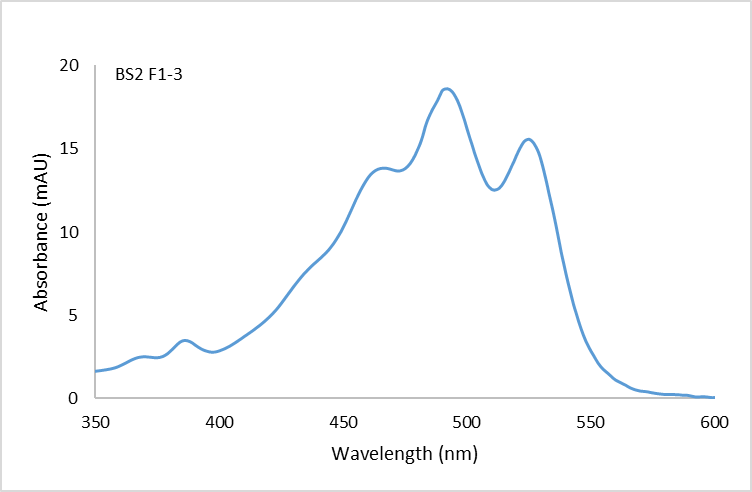

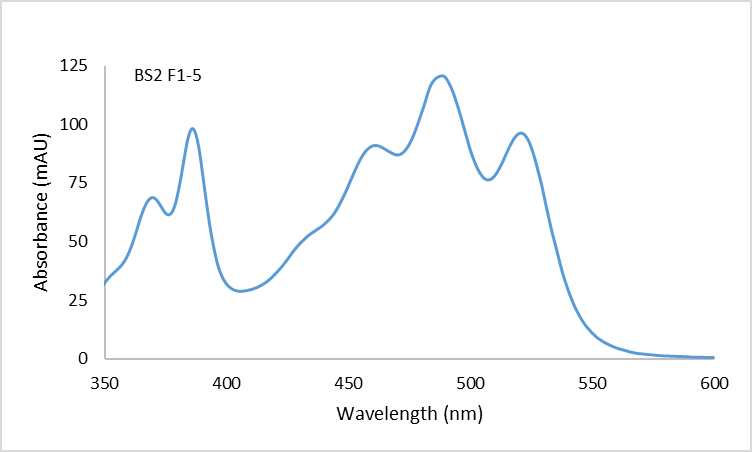

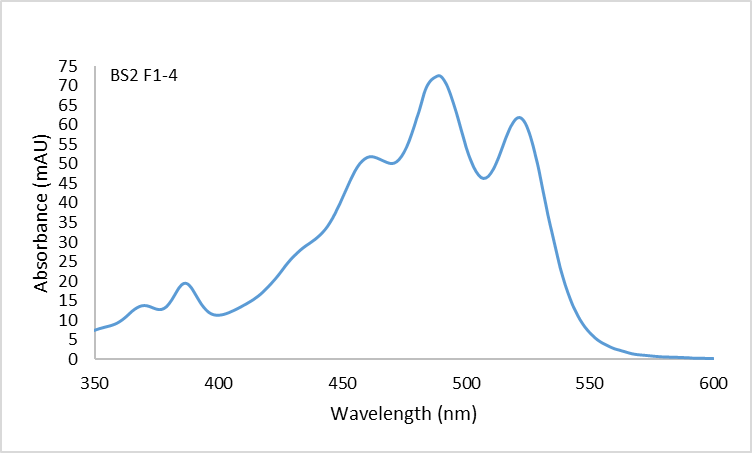


**Supplementary Fig 3**: Chromatographic, Mass and Spectrometric analysis of subfractions (F1-1 to 5) from fraction 1 (F1) of the carotenoid extract of Haloarcula rubripromontorii BS2


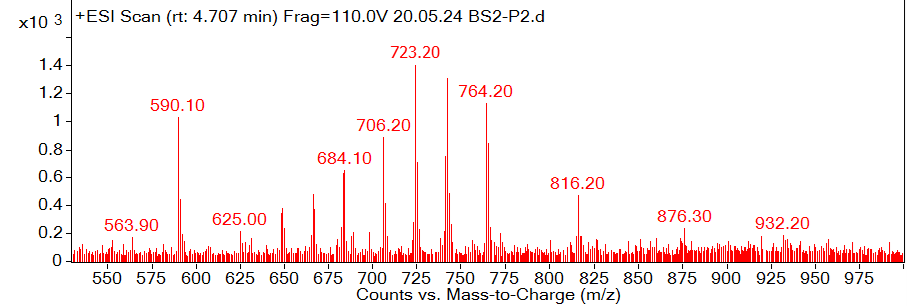

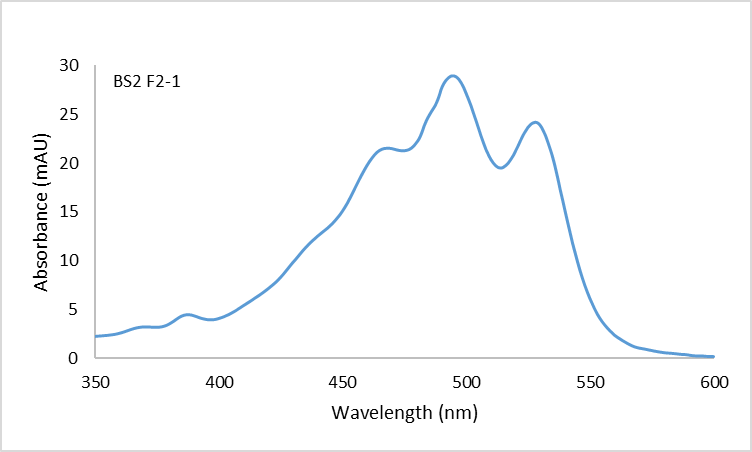

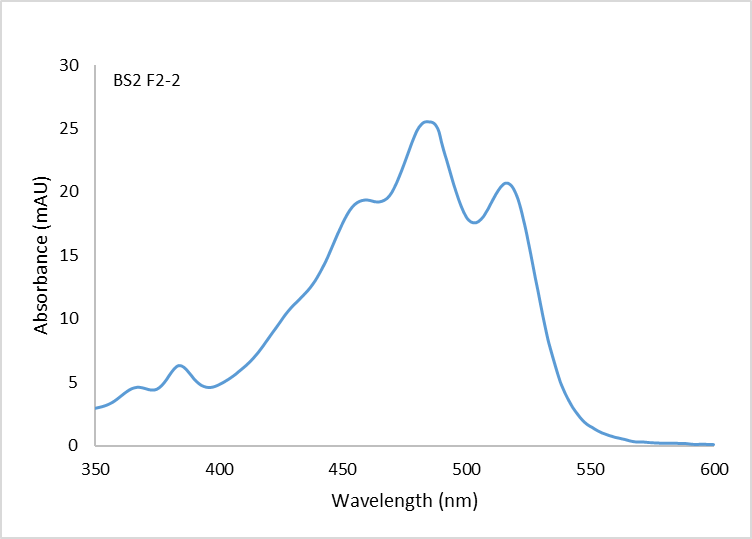

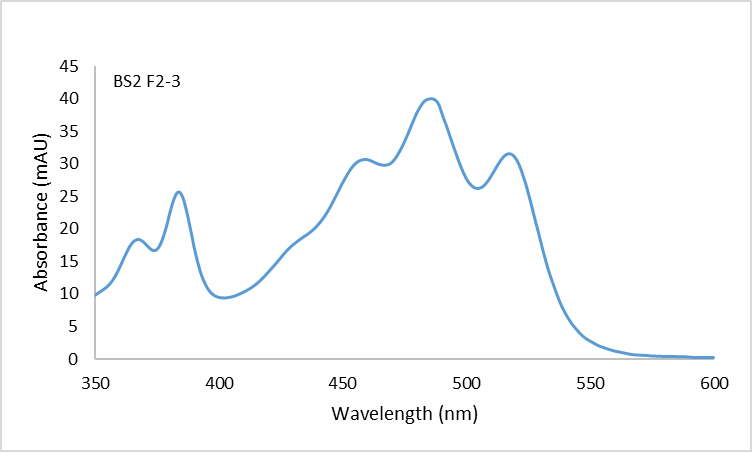

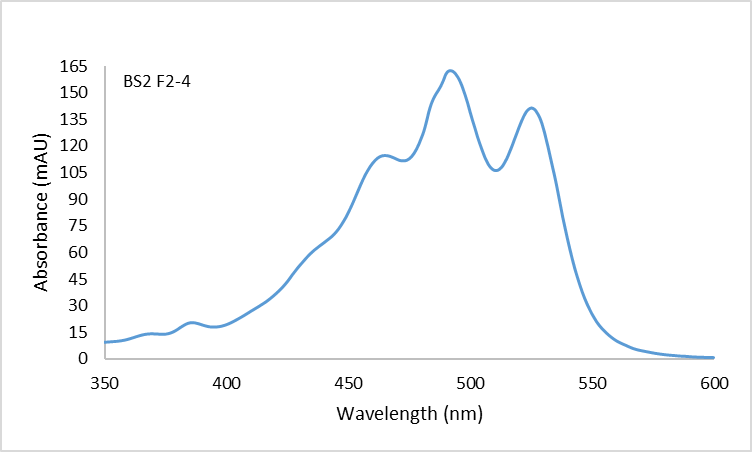


**Supplementary Fig 4**: Chromatographic, Mass and Spectrometric analysis of subfractions (F2-1 to 4) from fraction 2 (F2) of the carotenoid extract of Haloarcula rubripromontorii BS2

**Supplementary Fig 5:** Chromatographic, Mass and Spectrometric analysis of subfractions (F3-1 to 3 of fraction 3 (F3) from the carotenoid extract of Haloarcula rubripromontorii BS2


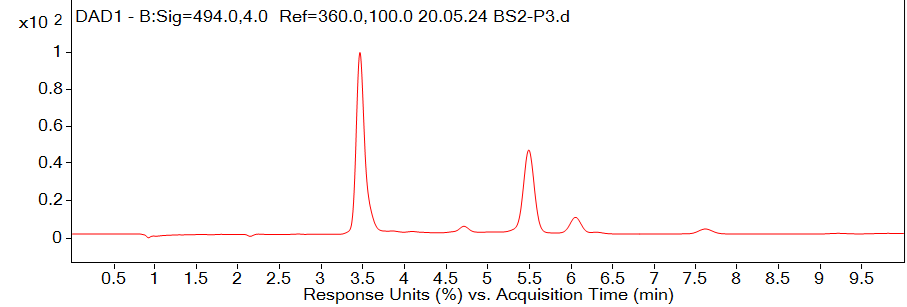

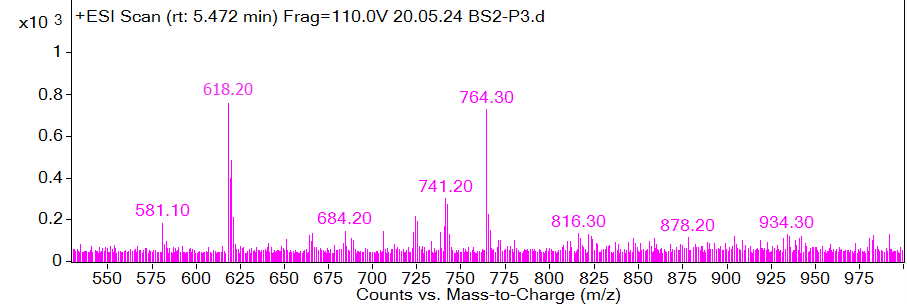

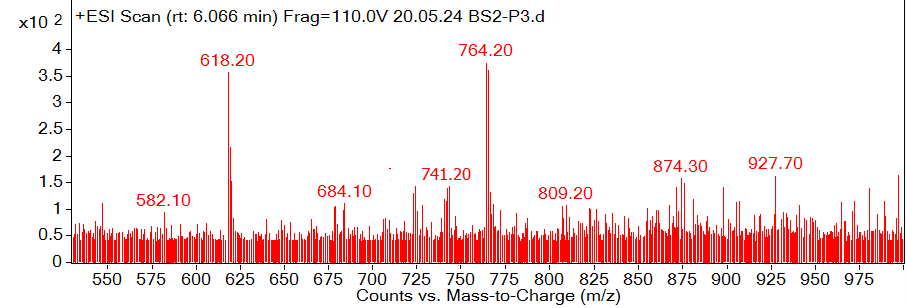

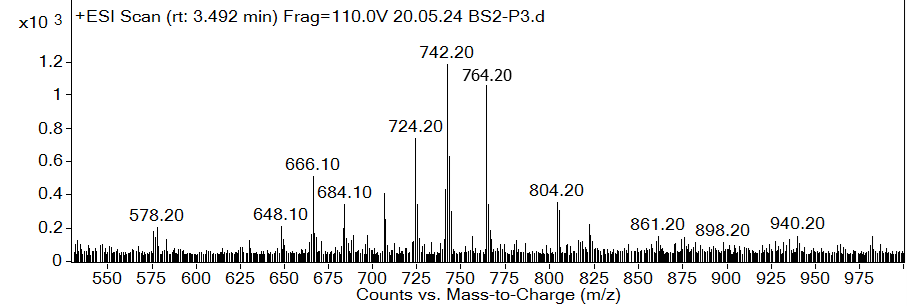

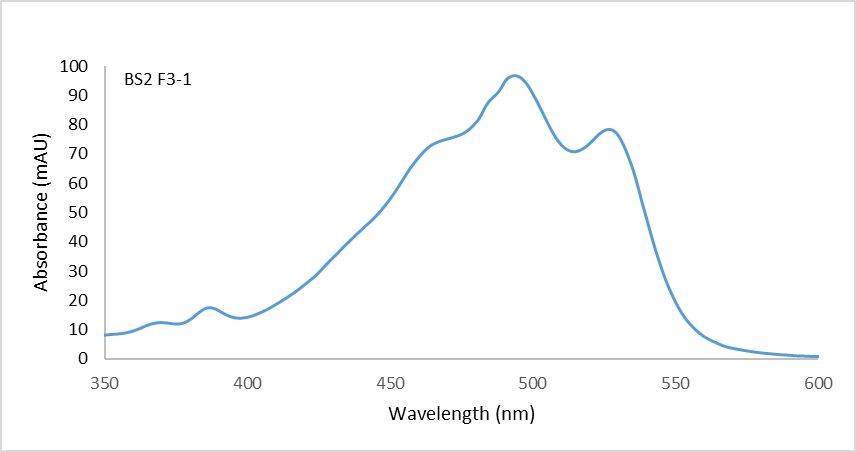

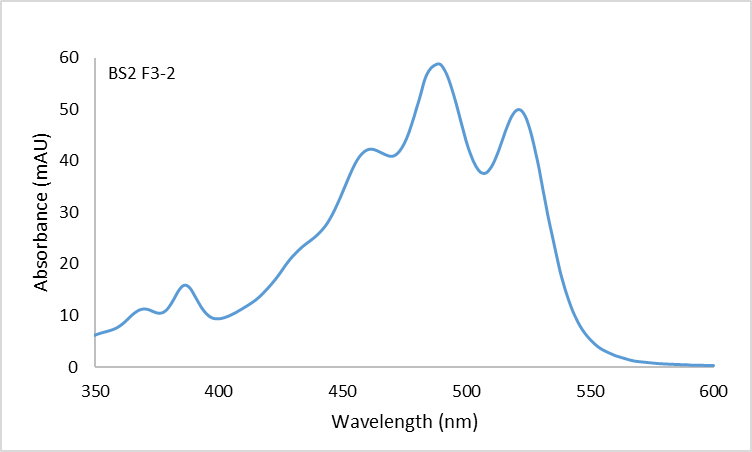

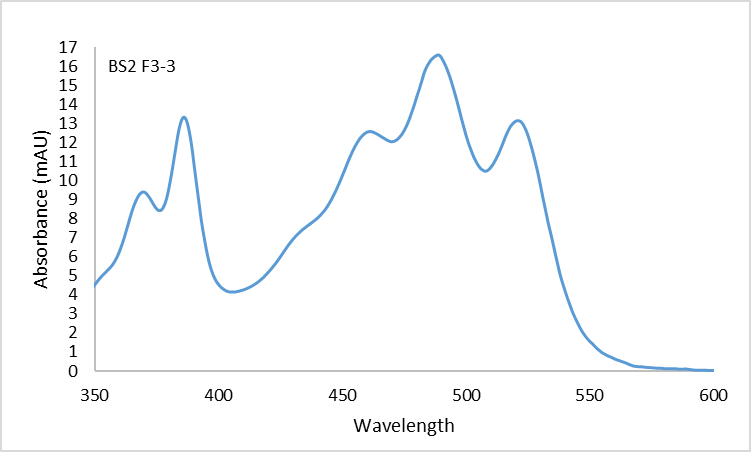


**Supplementary Fig 6:** Chromatographic, Mass and Spectrometric analysis of subfractions (F4-1 to 3) from fraction 4 (F4) of the carotenoid extract of Haloarcula rubripromontorii BS2


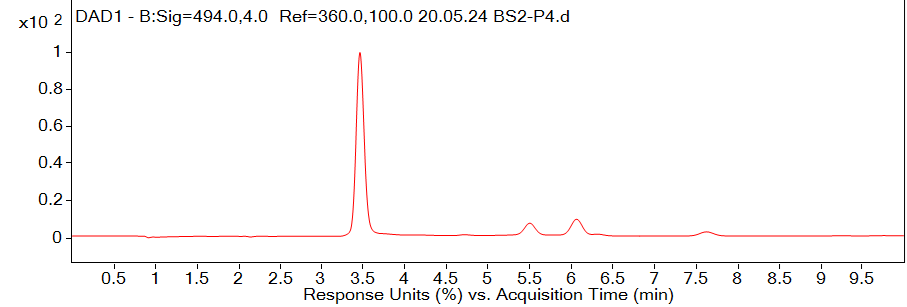

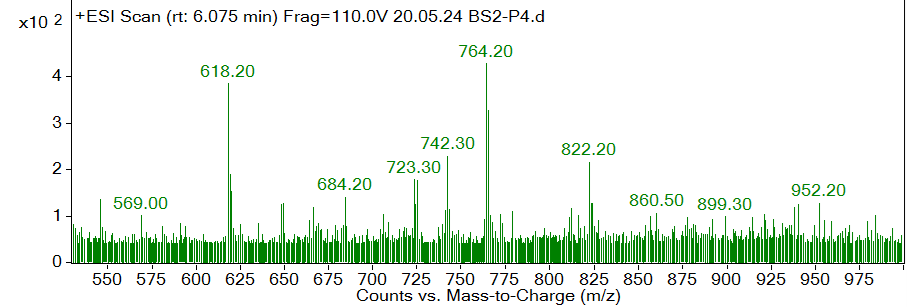

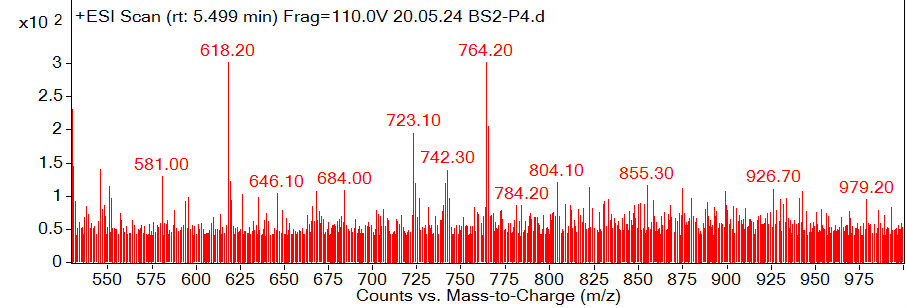

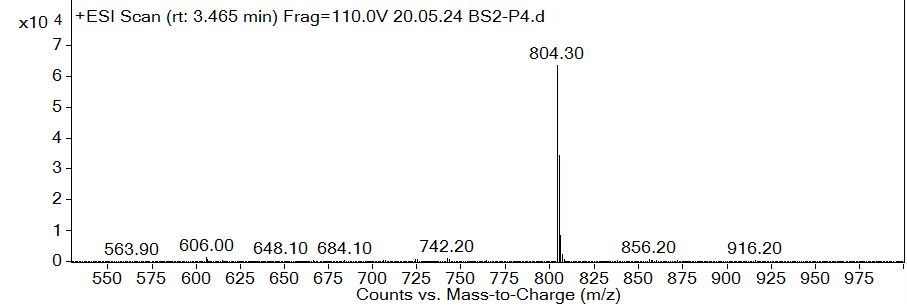

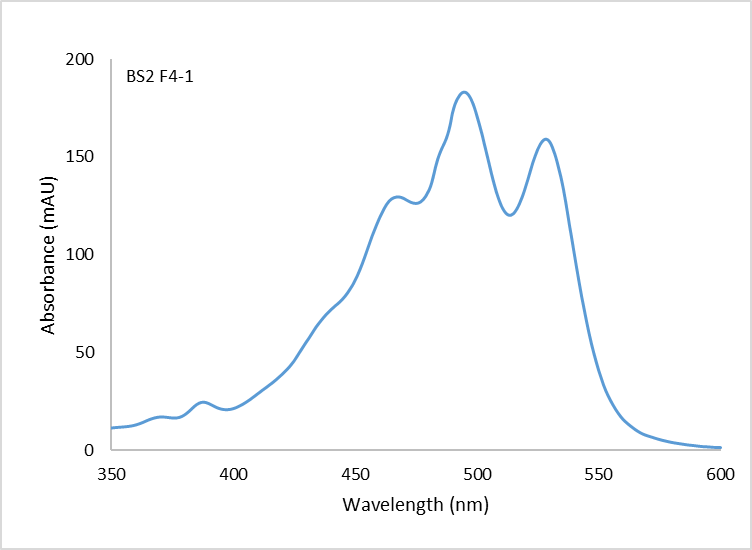

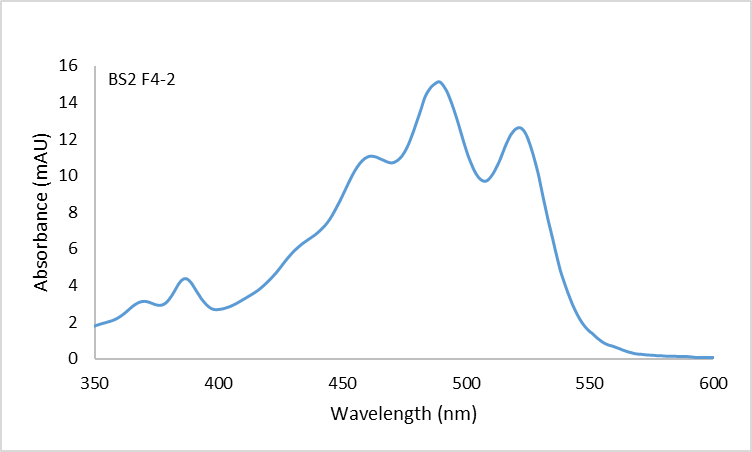

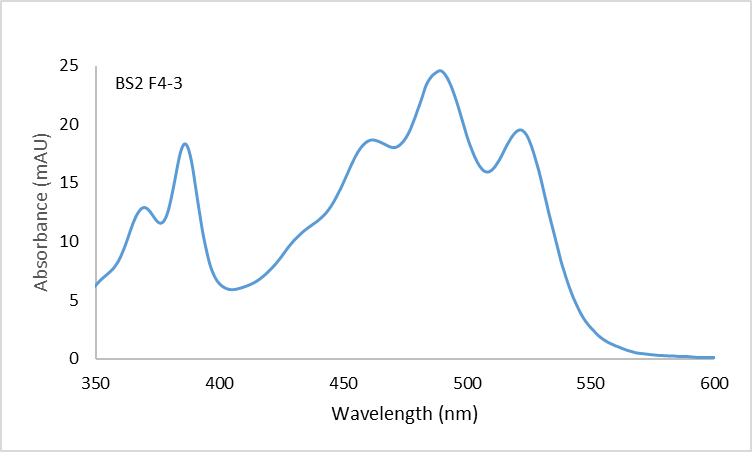


**Supplementary Fig 7:** Chromatographic, Mass and Spectrometric analysis of subfractions (F5-1 and 2) from fraction 5 (F5) of the carotenoid extract of Haloarcula rubripromontorii BS2


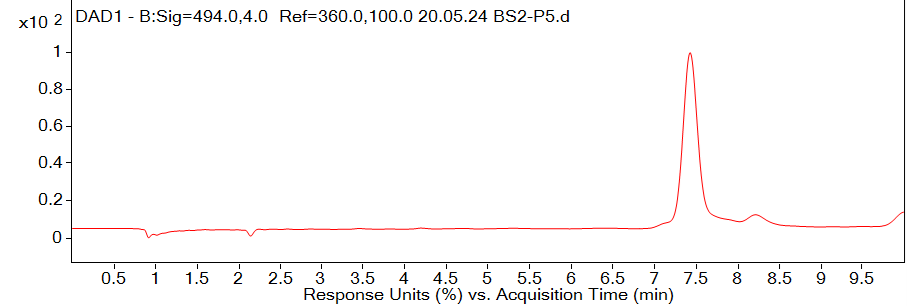

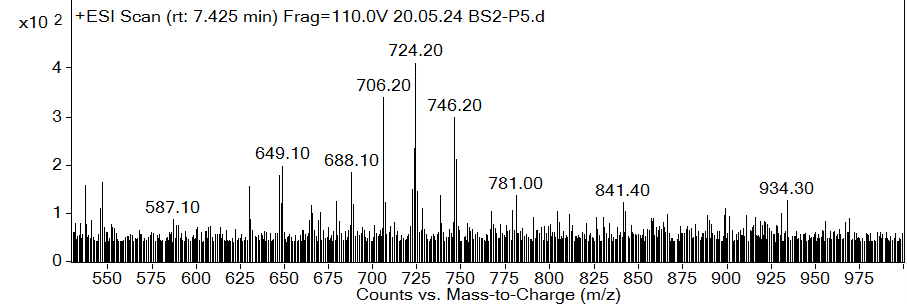

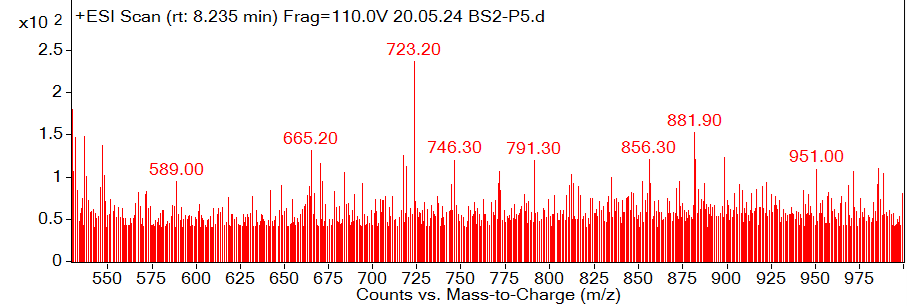

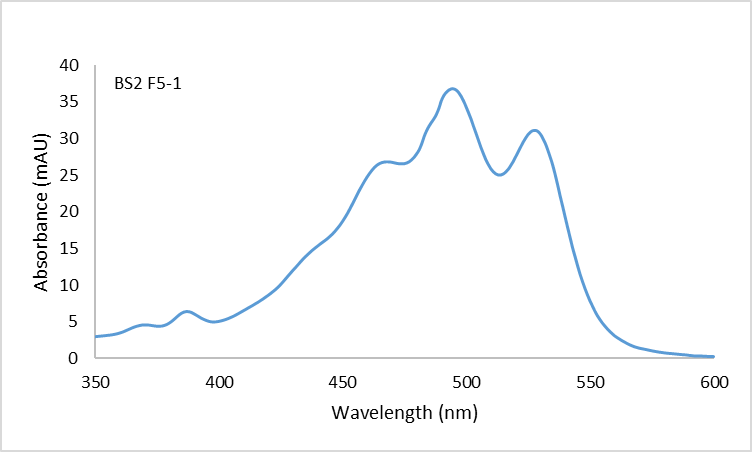

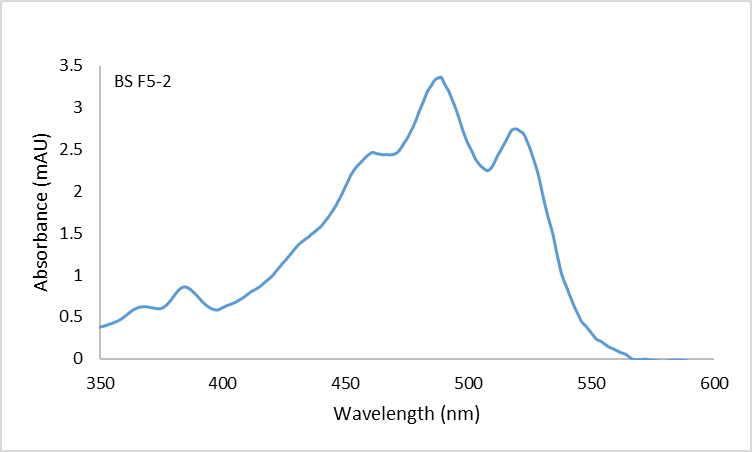

Supplement: Supplementary file 1 — Supporting Figure 1: TLC profile of the carotenoid extract of Haloarcula rubripromontorii BS2. Supporting Figure 2: UV‐visible spectra of the carotenoids from Haloarcula rubripromontorii BS2. Supporting Figure 3: Chromatographic, Mass and Spectrometric analysis of subfractions (F1‐1 to 5) from fraction 1 (F1) of the carotenoid extract of Haloarcula rubripromontorii BS2. Supporting Figure 4: Chromatographic, Mass and Spectrometric analysis of subfractions (F2‐1 to 4) from fraction 2 (F2) of the carotenoid extract of Haloarcula rubripromontorii BS2. Supporting Figure 5: Chromatographic, Mass and Spectrometric analysis of subfractions (F3‐1 to 3 of fraction 3 (F3) from the carotenoid extract of Haloarcula rubripromontorii BS2. Supporting Figure 6: Chromatographic, Mass and Spectrometric analysis of subfractions (F4‐1 to 3) from fraction 4 (F4) of the carotenoid extract of Haloarcula rubripromontorii BS2. Supporting Figure 7: Chromatographic, Mass and Spectrometric analysis of subfractions (F5‐1 and 2) from fraction 5 (F5) of the carotenoid extract of Haloarcula rubripromontorii BS2. [file MBO3-15-e70228-s001.docx]
